# Mass Customized Outlook for Regenerative Heart Failure Care

**DOI:** 10.3390/ijms222111394

**Published:** 2021-10-22

**Authors:** Satsuki Yamada, Jozef Bartunek, Atta Behfar, Andre Terzic

**Affiliations:** 1 Center for Regenerative Medicine, Marriott Family Comprehensive Cardiac Regenerative Medicine, Marriott Heart Disease Research Program, Van Cleve Cardiac Regenerative Medicine Program, Department of Cardiovascular Medicine, Mayo Clinic, Rochester, MN 55905, USA; yamada.satsuki@mayo.edu (S.Y.); behfar.atta@mayo.edu (A.B.); 2 Division of Geriatric Medicine and Gerontology, Department of Medicine, Mayo Clinic, Rochester, MN 55905, USA; 3 Cardiovascular Center, OLV Hospital, 9300 Aalst, Belgium; 4 Department of Physiology and Biomedical Engineering, Mayo Clinic, Rochester, MN 55905, USA; 5 Department of Molecular Pharmacology and Experimental Therapeutics, Department of Clinical Genomics, Mayo Clinic, Rochester, MN 55905, USA

**Keywords:** acellular, affordable, cardiopoietic, cardiopoiesis, clinical trial, cost, regenerative medicine, secretome, stem cells, therapy

## Abstract

Heart failure pathobiology is permissive to reparative intent. Regenerative therapies exemplify an emerging disruptive innovation aimed at achieving structural and functional organ restitution. However, mixed outcomes, complexity in use, and unsustainable cost have curtailed broader adoption, mandating the development of novel cardio-regenerative approaches. Lineage guidance offers a standardized path to customize stem cell fitness for therapy. A case in point is the molecular induction of the cardiopoiesis program in adult stem cells to yield cardiopoietic cell derivatives designed for heart failure treatment. Tested in early and advanced clinical trials in patients with ischemic heart failure, clinical grade cardiopoietic cells were safe and revealed therapeutic improvement within a window of treatment intensity and pre-treatment disease severity. With the prospect of mass customization, cardiopoietic guidance has been streamlined from the demanding, recombinant protein cocktail-based to a protein-free, messenger RNA-based single gene protocol to engineer affordable cardiac repair competent cells. Clinical trial biobanked stem cells enabled a systems biology deconvolution of the cardiopoietic cell secretome linked to therapeutic benefit, exposing a paracrine mode of action. Collectively, this new knowledge informs next generation regenerative therapeutics manufactured as engineered cellular or secretome mimicking cell-free platforms. Launching biotherapeutics tailored for optimal outcome and offered at mass production cost would contribute to advancing equitable regenerative care that addresses population health needs.

## 1. Introduction

### 1.1. Heart Failure Pandemic

Worldwide, sixty million people live with heart failure today, stressing the ongoing burden of cardiovascular disease on modern society [1,2,3]. Affecting two percent of adults, heart failure develops in one of five individuals during their lifetime. This alarming epidemic is anticipated to worsen as the global population continuously grows older, contributing to a sustained healthspan–lifespan gap, further compounded by a younger, increasingly more morbid population at risk of developing heart failure [4,5,6]. Chronic heart failure syndrome is malignant in its progressive non-reversible nature, and notoriously refractory to treatment options resulting in recurrent hospitalizations and poor survival causing death in two-thirds of patients within five years of diagnosis [7]. Pump failure is a common manifestation of florid disease shared across heart failure etiologies, provoked by complex underlying cardiomyopathic substrates [8]. Molecular insights into the disease pathophysiology empower the search for targeted solutions, helping secure an expanded armamentarium aimed at supporting longitudinal wellness. Accordingly, the development of tailored strategies that adequately restore organ structure and function is an area of vigorous investigation in cardiovascular medicine [9,10].

### 1.2. Emerging Strategies

The standard-of-care for chronic heart failure incorporates lifestyle change, pharmacotherapy, device implantation, and transplantation [11,12]. Treatment choices are characteristically limited to symptom mitigation in the course of disease and ultimately palliative at the terminal stage [13]. Notwithstanding, advanced heart failure management remains restricted by a persistent shortage of available donor organs, stringent criteria for transplantation, and complications associated with life-extending durable mechanical support options [14,15]. Ideally, innovative treatments would be proactive rather than reactive, intended to limit organ deterioration and reverse disease progression to avoid the need for advanced therapeutic interventions [16]. In this context, mechanism-based, translatable targets for precision healthcare solutions would complement presently available strategies. The introduction of patient-centric regenerative technologies exemplifies an emerging class of disruptive innovation designed at achieving normative organ restitution [17,18]. The spectrum of nascent regenerative biotherapies is conceived practice transformative, aspiring to rebuild health. Effective translation of regenerative science thus ushers the prospect of a “care to cure” evolution in disease management, poised to evolve the patho-demographic landscape.

## 2. Regenerative Paradigm

### 2.1. From Promise to Reality

#### 2.1.1. Core Principles

Regenerative therapies are assumed to boost an otherwise limited capacity of the adult heart for self-repair [19]. Permissive toward regenerative interventions, the failing heart is an acknowledged candidate for restorative procedures [20]. Advances in the science of developmental biology, biomedical engineering, and nanomedicine have enabled the growth of a diverse regenerative toolkit [21,22,23,24]. In particular, stem cells and respective derivatives have generated substantive clinical experience while raising the expectations of patients and the public alike [25,26,27]. Across the span of the last two decades, cardiovascular trials have tested a range of cell products including mononuclear, mesenchymal, cardiac, and more recently pluripotent stem cells [28,29,30]. Stem cell therapy was originally envisioned as a means to directly rebuild an ailing heart muscle. More recent considerations implicate an indirect, paracrine-mediated mode of action. Myocardial repair would reflect the interaction between the regenerative potency of a delivered cell product and the intrinsic aptitude of the failing heart microenvironment to answer through a healing response [31,32,33]. These recent advancements in the understanding of regenerative biology underscore the significance of mobilizing inherent reserves in support of rebuilding heart tissue health.

#### 2.1.2. Current Experience

Bench-to-bedside translation mandates a rigorous safety and efficacy evaluation in pre-clinical testing. The process pertinent to stem cell-based interventions, and applicable in cardiovascular medicine, has been outlined in guidelines by the International Society for Stem Cell Research and the European Society of Cardiology Working Group on Cardiovascular Regenerative and Reparative Medicine [34,35]. In accordance with Good Laboratory Practice Standards, recommendations to improve the quality of pre-clinical research that justify undertaking clinical trials in humans include: (i) blinding; (ii) randomization; (iii) pre-study definition of inclusion and exclusion criteria; (iv) pre-reporting of protocol design and study rationale, parameters, and readouts; and (v) evidence of independent corroboration with the purpose to maximize transparency and reproducibility, and reduce potential bias [35]. In cardiac regenerative therapy, pre-clinical investigation has reached over a thousand distinct studies employing multiple combinations of biologics, disease models, utilized species, delivery routes, and dosing regimens [36]. Pre-clinical work, as of today, has led to 793 registered clinical trials categorized under the keywords of “heart” and “cell therapy” at the U.S. National Library of Medicine database, ClinicalTrials.gov. This experience, based on over 1700 pre-clinical and clinical trials, has collectively enabled increasing levels of evidence with reassuring feasibility and safety for adult stem cell use [37,38,39,40,41,42,43]. Indicators of benefit, however, have remained inconsistent among treated patients [44,45]. The recognized non-uniform bioactivity of stem cell populations triggers heterogenous clinical responses [35]. Standardizing regenerative potency before intervention in a way that reflects the product’s relevant biological properties is therefore necessary to ensure more reliable outcomes.

### 2.2. Addressing Reliability

#### 2.2.1. Optimizing Tactics

Attempts to enhance the reliability and achieve a desired therapeutic outcome have included multiple tactics focused on the regenerative product itself and its delivery, including habituation to the host environment, matching of the cell source with the recipient organ, use of combined therapy for synergistic action, or securing enhanced engraftment [46,47,48]. Beyond traditional stem cells, applied in their native or largely unmodified form, the quest for a dependable cardio-regenerative cell type has led to the generation of their fit-for-purpose derivatives, pivoting away from a generic biologic source into highly customized alternatives [49,50].

#### 2.2.2. The Cardiopoiesis Option

The regimented imposition of the “cardiopoiesis” program illustrates an optimizing strategy developed to promote the cardioreparative aptitude of delivered cells [51]. In this way, lineage-specifying instructions, applied by virtue of a conditioning growth factor cocktail, guide transition of a naïve stem cell phenotype into a cardiopoietic counterpart with defined transcriptome dynamics and enhanced therapeutic features [52]. Through molecular mimicry of embryonic signals that instruct the pre-cardiac mesoderm, a set of recombinant proteins or regulators, namely TGF-β, BMP-4, Activin-A, IGF-1, IL-6, FGF-2, thrombin, and retinoic acid, induce cardiopoiesis in human adult stem cells [51,52]. Applied to the mesenchymal cell type, co-stimulation with TGF-β, BMP-4, Activin-A, plus retinoic acid provokes cytosolic expression of cardiac transcription factors, IGF-1 and IL-6 instigate nuclear translocation, and FGF-2 with thrombin ensure maintained cell cycle activity. Recombinant growth factors facilitate cardiopoiesis, disrupting the latent plasticity of adult stem cells and stimulating cardiovasculogenic programming within a sustained proliferative state. Compared to lineage-unspecified mesenchymal cells, their cardiopoietic progeny display an improved therapeutic impact when tested in failing hearts [51]. Gene expression profile-based release criteria have accordingly been developed as a quality standard measure to pre-assess the regenerative fitness of patient-derived stem cells [53]. Such a “cardiopoietic index” reflects an integrated molecular readout, based on the mRNA expression of cardiogenic transcription factors, including Nkx2.5, MEF2c, Gata-4, MESP1, and Tbx5, thereby leveraging successful induction of cardiopoiesis as a gauge of anticipated therapeutic benefit [54].

## 3. Clinical Trial Experience

### 3.1. C-CURE and CHART-1 Clinical Trials

Enabled by pre-clinical studies and standardized operating procedures [55,56], clinical grade cardiopoietic cells have been tested in early and advanced trials (Table 1). The prospective multi-center Phase 2 C-CURE trial (Cardiopoietic stem Cell therapy in heart failURE) implemented lineage guidance in cell therapy [57]. Patients with ischemic heart failure were randomized to receive standard-of-care with versus without lineage-specified adult cardiopoietic cells. Of autologous, bone marrow-derived mesenchymal stem cell origin, these cells were delivered endomyocardially using electromechanical mapping. Cardiopoietic cell therapy was found feasible and safe, with signs of benefit additive to standard-of-care and measured by improved left ventricular ejection fraction, reduction in left ventricular end-systolic volume, and enhanced exercise capacity on the 6 min walk distance test (Table 1). The Phase 3 CHART-1 trial (Congestive Heart failure cArdiopoietic Regenerative Therapy) was the largest clinical trial in ischemic heart failure that assessed outcomes up to 2 years following a single dose of adult cardiopoietic cells delivered with a retention-enhanced endomyocardial catheter [58,59,60,61]. The CHART-1 trial was executed across 39 clinical centers in 10 countries, with 315 patients on standard-of-care and randomized 1:1 to receive cardiopoietic cell therapy or sham (Table 1). Clinical follow-up documented that cardiopoietic cell therapy is safe overall. While the untargeted ischemic heart failure population showed a neutral readout, post hoc analysis suggested sustained benefit in reducing risk of death or heart failure hospitalization in target patient subpopulations defined by the degree of left ventricular enlargement (left ventricular end-diastolic volume 200–370 mL) and tolerable cell dosing (≤19 injections) [61].

**Figure 1:**
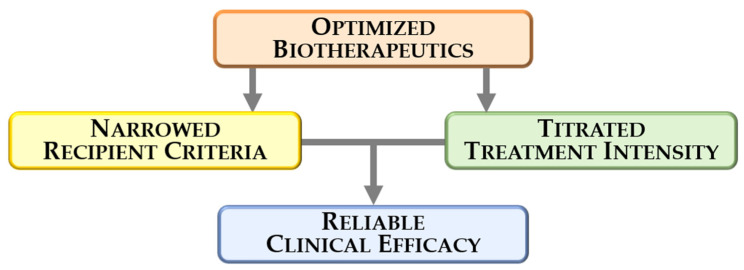
Contributors to the clinical benefit of a regenerative therapy include: (i) use of optimized biotherapeutics with predictable therapeutic potency pre-assessed prior to delivery; (ii) selection of candidates most likely to respond to therapy; and (iii) delivery of adequate treatment dosing.

Post hoc analyses should be considered exploratory and hypothesis-generating. The consistency in outcomes across the longitudinal experience and the continued clinical benefit driven by the accrual of relevant endpoints through the 104 weeks of follow-up warrant additional investigation and validation. Indeed, the long-term follow-up offers guidance for future targeted trials. In this context, the Food and Drug Administration granted a “Fast Track” designation to cardiopoietic cell therapy for reduction in mortality, hospitalization, and improvement in quality of life for patients with chronic heart failure secondary to advanced ischemic cardiomyopathy [62]. Collectively, clinical experience with cardiopoietic cell therapy introduces a regenerative product optimized pre-delivery and informs the assessment of targetable recipient populations (Figure 1).

### 3.2. Reverse Translation

When tested in a highly controllable research setting, hypotheses raised by clinical experience, including expected or unexpected outcomes, enable systematic validation and exploration of potential applications. Case in point, disease severity is observed to impact the outcome of cardiopoietic cell therapy [58,61], with the relationship between left ventricular size and functional/structural restitution post-therapy confirmed in a disease model [63]. Specifically, the best responders to intramyocardial delivery of cardiopoietic cells were among recipients presenting a left ventricular end-diastolic volume equivalent to 200–370 mL in the human heart. Smaller or larger hearts were less responsive. Such “just right” pattern of a ventricular size dependent response implicates a “Goldilocks principle” for benefit following cardiac regenerative interventions [63]. Indeed, left ventricular size is a marker of organ remodeling with a window in disease progression when the failing heart has not passed the point of no return, and is most amenable for therapy [64,65]. Accordingly, reversal of left ventricular enlargement serves as a surrogate of therapeutic success [66,67,68]. While initially described with cardiopoietic cell therapy in ischemic heart disease and pinpointed at the molecular level on the basis of documented reverse remodeling of the cardiac proteome [63], a left-ventricular-size-dependent outcome was also documented in non-ischemic heart failure treated with distinct cell types [69]. Disease severity should therefore be considered in the design of patient-specific clinical markers, potentially informing the selection of the most suitable candidates to receive cell therapy.

### 3.3. Clinomics Approach

The emergence of “clinomics” deepens fundamental knowledge of disease mechanisms, leveraging readouts of effectiveness in clinical trials. Recent technological advances have led to a rapid deployment of a high-definition arsenal that facilitates the implementation of big data-driven precision medicine [70,71]. Namely, applied multiomics include the exploration of the genome, transcriptome, proteome, immunome, metabolome, or microbiome. In conjunction with longitudinal clinical and behavioral phenotyping, ‘omics technologies have the potential to advance the science of novel biotherapies (Table 2).

**Figure 2:**
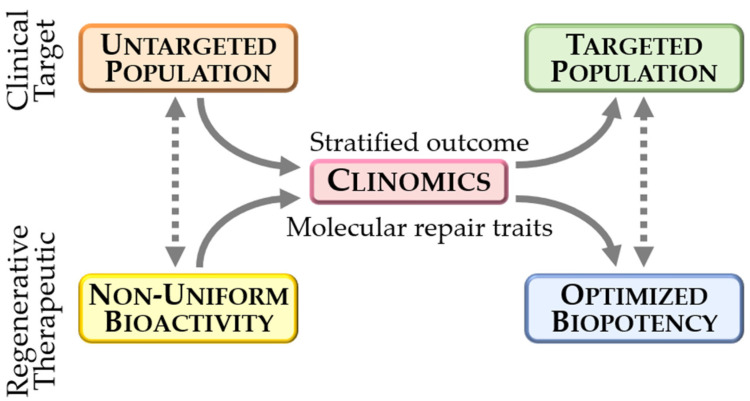
Convergence of new knowledge distilled from clinical trials and regenerative science has expedited the stratification of responders versus non-responders to biotherapy while decoding the molecular underpinnings associated with repair capacity. The era of “clinomics” leverages clinically available biospecimens, including clinical trial biobanked stem cells, to help resolve through ‘omics methodologies the core components underlying therapeutic bioactivity. Iterative, integrated analysis guides the development of next generation biotherapeutics endowed with regenerative biopotency and optimized to achieve consistent outcomes in defined candidate populations.

In this way, clinical trial biobanked cells have helped characterize the intimate features of cell-based cardiac repair. By profiling the molecular influence on recipient hearts, high throughput systems biology revealed that cardiopoietic cell therapy transitions diseased hearts from their cardiomyopathic trajectory toward pre-disease [72]. The cardiac ventricular proteome exhibits extensive molecular remodeling imposed by chronic disease yet retains malleability, enabling disease course reversal in response to stem cell therapy. Cardiopoietic cell intervention rectified the disease comprised molecular substrate, substituting cardiomyopathic with reparative attributes of vasculogenesis, cardiac development, and organ regeneration. It is yet to be determined whether cardiopoietic cell-mediated molecular restoration, characterized by a non-random reversal of disease-perturbed molecular derangements, is unique to therapy with this lineage-guided cytotype or is rather shared across regenerative biotherapies. While delivered stem cells, including cargo that they release, are the presumed active ingredient, the mode of action remains to be fully defined. Notably, documented efficacy despite limited integration of delivered cells into the recipient organ supports a proposed paracrine contribution [73,74]. By leveraging high versus low response clinical cohorts, clinomics-based interrogation of the composition and functionality of the differential cardiopoietic cell secretome linked therapeutic fitness to inherent vasculogenic properties, along with cardiac and smooth muscle differentiation and development [74]. Distinguishing cardiopoietic cells endowed with enhanced therapeutic capacity, the resolved (cardio)vasculogenic secretome integrated with a distinct intracellular microRNA profile, in accord with the centrality of microRNA systems in regulating regeneration [74]. Notably, the downregulated microRNA-146 cluster linked to a protein directed network, characterized by the activation of NFkB, STAT1/6 and CREB1 transcription pathways [74]. The microR-146-dependent system encompassed enrichment consistent with prioritized cardio-vasculogenesis of the reparative secretome. Mirroring the secretome pattern, infarcted hearts with a high response to therapy displayed a reformed myocardial proteome distinguished by an enhanced aptitude to engage across enriched cardiovascular system functions [74]. Molecular profiling of the stem cell secretome may thus offer predictive value in selecting proficient regenerative biologics, paving the way for the development of an increasingly optimized biotherapeutic toolbox. Enlightening the molecular basis that underpins clinical outcomes thus provides an inroad to identify determinants of prospective benefit, informing the success of a novel therapy. Clinomics can thus provide relevant information for advancing precision regenerative care (Figure 2).

## 4. Efforts toward Optimization

### 4.1. Scalable Cost-Effective Therapy

Sustainable use of stem cells in practice is challenged by high cost, requiring cost-effective measures, including scalable and standardized procedures. With the need for mass customization, efforts are under way to ensure cost savings in tandem with uniformity of therapeutic impact.

#### 4.1.1. Single Gene Engineering of Repair Competent Cells

Illustrating the means to bypass avoidable steps in cell production, cardiopoietic guidance has been streamlined from the demanding, recombinant protein cocktail-based protocol to a protein-free, messenger RNA-based single gene transfection to engineer affordable cardiac repair competent cells [75]. To this end, microencapsulated-modified-messenger RNA (M3RNA) technology has achieved targeted gene delivery through nonintegrating and viral-free transfection [76]. Permutations of mesodermal and precardiac transcription factors, delivered in isolation or in combination by the M3RNA-based gene transfer system, delineated a single gene, namely Brachyury, suitable and sufficient for induction of cardiopoiesis. Brachyury is an established master regulator of mesoderm development, primordial in earliest cardiovascular differentiation. Brachyury was proficient in yielding vasculogenic, antioxidant, and immunomodulatory cell properties, with an antifibrotic outcome and rescue of the heart failure syndrome following intramyocardial delivery [75]. In principle, by overcoming the taxing nature of protein cocktail-based cardiopoiesis experienced with autologous populations, molecular engineering could enable a minimalistic and scalable approach for pre-delivery optimization of an allogeneic regenerative cell product.

#### 4.1.2. Distilled Essence for Cell-Free Regeneration

The cell-centric focus has been steadily broadened by alternative approaches for the development of biotherapeutics, reflecting the maturation of regenerative technologies. As an alternate to traditional cell use, consideration of cell-free approaches is supported by resolving the defining cell secretome properties associated with therapeutic benefit [77]. Zooming in on the actionable target of therapy is at the core of “acellular” regenerative science, facilitated by the exploration of secreted extracellular vesicles, denoted “exosomes” and implicated in cell-to-cell communication [78,79]. Derived exosomes, instead of the stem cell source, has the potential to harness relevant cell therapy features while concentrating the resolved active ingredient to endow flexibility of dosing biopotency in a ready-to-use product [80,81]. Properly purified and formulated exosome-based therapies would help remove some of the issues, such as cell survival and unstable bioactivity, that impede cell-based approaches and could simplify the infrastructure-intense processes typically needed in the production and clinical delivery of cellular therapeutics [81]. If derived in sufficient quantity without the cost burden of cell manufacturing, the knowhow carried from the stem cell-centric platforms could be translated into acellular, active ingredient enriched, alternatives apt to provide consistent performance at lower cost. Notably, non-cellular biologics would streamline regenerative therapy applications rendering it increasingly feasible for point-of-care, and therefore no longer limited to tertiary care centers.

### 4.2. Toward Mass Customization

#### 4.2.1. Forward Translation

Whether delivered in the form of a cellular or an acellular product [77], establishing an evidence-based regimen requires a close evaluation of regenerative treatment protocols (e.g., singular versus repeat interventions, with or without adjuvant support) both from a medical as well as from an economic perspective. Formulation of regenerative therapeutics into next generation cell engineered or cell-free counterparts can profoundly impact pharmacokinetic and pharmacodynamic attributes but also facilitate product manufacturing, delivery, and overall reliability, generating cost-effective, broadly available treatment solutions [80]. Cell engineering strategies could help overcome cell-to-cell variability in regenerative potency inherent to autologous cell therapies by producing made-to-order allogeneic products at mass production cost [81]. Similarly, cell-free products mimicking the paracrine impact of cell-based therapies would enable an off-the-shelf availability and standardized dosing [78,79]. The path to adoption in cardiology care will thus mandate a transdisciplinary effort bringing together multiple specialties to establish validated and fiscally responsible regenerative therapy guidelines across the discovery–development–delivery continuum [82,83]. In parallel with the focus on optimizing the regenerative product and associated procedures, standardization warrants the narrowing of “inter-patient” variability linked to the genetic make-up, comorbidities, disease pathobiology, and innate variance in responsiveness reflective of diversity among candidate recipients [84].

#### 4.2.2. On-Demand Solutions with Expanded Access

**Figure 3:**
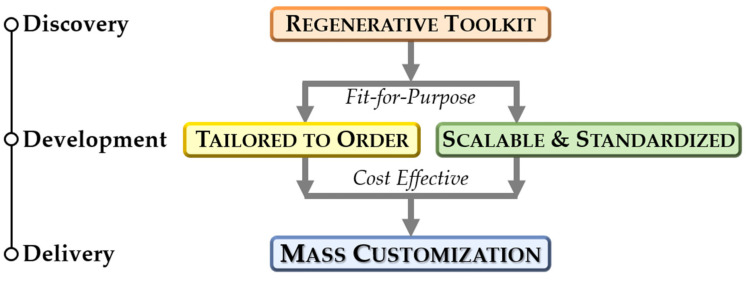
Mass customization, an imperative in attaining sustainable and democratized regenerative care, requires a fit-for-purpose streamlining of a diverse innovation toolbox to develop a cost-effective supply of made-to-order, scalable, and standardized regenerative products.

Clinical trials are valuable in establishing safety and efficacy in phenotypically defined cohorts. However, there is a recognized gap between trial scenarios and real-world care. In contrast to the “one-size-fits-all” mass production approach used by traditional pharmaceutical paradigms, the idiosyncrasy associated with regenerative biotherapies mandates mass customization, calling for on-demand solutions tailored to ever-changing needs while ensuring broad at-cost distribution [85,86]. Flexible to overcome undesirable variations, this demand-driven model is poised to achieve better patient outcomes and advance therapeutic innovation for accessible benefit of populations in need (Figure 3). To this end, the regenerative ecosystem must integrate: (i) at the research level, deeper characterization of the regenerative mode of action; (ii) at the translational level, advanced manufacturing for mass production of tailored biologics; (iii) at the clinical level, better segmentation of the pathophenotype to guide individual care; and (iv) at the post-approval adoption level, patient/public delivery informed by cost-effectiveness.

### 4.3. Validity and Utility

Science-driven responsible translation of innovative technologies is a cornerstone in establishing new care pathways. Clinical development has marshaled regenerative therapeutics while integrating a multivalent assessment reflective of standards set by providers, developers, regulators, and payers to advance a curative model of care [87]. Clinical readiness mandates achieving both “validity” (documented effectiveness) and “utility” (improved outcomes) to provide a value-added benefit for patients and healthcare systems [88]. A regenerative technology-empowered model of care, with a robust supply chain, advanced access and added therapeutic value, requires comprehensive evidence amassed through the development process adhering to rigorous quality control and regulatory compliance [89]. Delivery, meeting stringent ethical norms, is predicated on trained healthcare professionals educated to achieve the highest degree of proficiency in practicing regenerative care [90]. Moreover, complementing technology readiness, institutional readiness—whereby healthcare systems have reached the capacities needed to adopt economically viable innovation—is a prerequisite for achieving the imperative of long-term sustainability [91,92]. Indeed, the collective resolve to advance regenerative science breakthroughs into daily clinical practice aspires to realize health benefits for all [93].

## 5. Conclusions

Heart failure is in the top 5% of the most expensive medical costs, with care programs unable to impact rampant expenditure [94]. Projected to alter the healthcare future, the advent of high-definition medicine offers refined assessment and management of human health at an unprecedented resolution [95]. The convergence of life and material sciences has remarkably advanced the development of next generation biotherapies for heart failure [96]. Accelerated by the decryption of mechanisms underlying left ventricular dysfunction, novel treatment options are actively tested in clinical trials documenting a disease-modifying bioactivity that complements standard-of-care [97,98]. However, there is a recognized challenge in translating trial results into value for clinicians and patients, as lifetime benefits for new heart failure treatments are yet to be established [99,100]. In considering the viability of regenerative medicine regimens, an increased emphasis is placed on assessing the validity and potential utility of newly optimized biotherapeutics coupled with a better pre-selection of treatment candidates. Beyond ensuring reproducible safety and efficacy across patient populations, clinical translation mandates careful attention of the value-based proposition compatible with routine care [101,102]. Scalable, mass customized products are ultimately needed to realize a sustainable, cost-effective prospect, enabling a social outlook of highly accessible and affordable regenerative medicine solutions. Within the 2030 horizon, achieving proven and broadly reachable regenerative therapies will be paramount in ensuring an equitable coverage for at-risk populations.

## Figures and Tables

**Table 1:** Multi-center randomized clinical trials assessing cardiopoietic cells for chronic ischemic heart failure.

	C-CURE	CHART-1
Phase	Early (phase 2) trial	Advanced (phase 3) trial
Endpints	Feasibility and safety	Efficacy using hierarchical composite
	Cardiac function/structure	Safety
	Global clinical performance	
Participants	47 pts screened and randomized	315 pts met criteria and were randomized into
	15 SOC (control)	158 SOC plus sham procedure (control)
	21 SOC plus cell therapy	157 SOC plus cell therapy
Follow-up	6 months	Up to 104 weeks
Readouts	Feasibility/safety endpoints	At 39 weeks [58]
	75% success in manufacturing	Neutral across the whole cohort
	100% success in cell delivery	Benefit in pts with baseline LVEDV 200–370 mL
	No cardiac/systemic toxicity	No difference in serious adverse events
	Documented safety profile	Aborted or sudden cardiac death in 5.4% pts without and in 0.9% pts with cell therapy
	Efficacy endpoints	
	Improved LVEF	At 52 weeks [59]
	Reduced LVESV	Reduced LVEDV and LVESV
	Improved 6 min walk distance	At 104 weeks [61]
		Neutral in the whole cohort
		Reduced risks of death or hospitalization in subcohort with baseline LVEDV 200–370 mL treated with ≤19 injections
		No difference in safety readouts

Cardiac events: death, elective transplant and arrhythmias, C-CURE: Cardiopoietic stem Cell therapy in heart failURE [57], CHART-1: Congestive Heart failure cArdiopoietic Regenerative Therapy [58,59,61], hierarchical composite: all-cause mortality, worsening heart failure, Minnesota living with heart failure questionnaire score, 6 min walk distance, left ventricular ejection fraction (LVEF), and left ventricular end-systolic volume (LVESV), LVEDV: left ventricular end-diastolic volume, pts: patients, SOC: standard-of-care, vs: versus.

**Table 2:** Leveraging clinical trials, clinomics aim to achieve a comprehensive, deep phenotyping using a high precision, high throughput toolkit.

	Clinical Phenotyping	Molecular Phenotyping
Goal	Safety and efficacy	Mechanism of action
Datasets	Small to moderate	Large
Analysis	Manual	AI integrated
Readouts	Demographics	Genome
	Physical examination Risk stratification Laboratory tests Imaging Catheterization	Transcriptome Proteome Immunome Metabolome Microbiome

AI: artificial intelligence.
